# Effect of Water and Glycerol in Deoxygenation of Coconut Oil over Bimetallic NiCo/SAPO-11 Nanocatalyst under N_2_ Atmosphere

**DOI:** 10.3390/nano10122548

**Published:** 2020-12-18

**Authors:** Rungnapa Kaewmeesri, Jeeranan Nonkumwong, Thongthai Witoon, Navadol Laosiripojana, Kajornsak Faungnawakij

**Affiliations:** 1 The Joint Graduate School of Energy and Environment (JGSEE), King Mongkut’s University of Technology Thonburi, Bangkok 10140, Thailand; rungnapa@nanotec.or.th (R.K.); navadol@jgsee.kmutt.ac.th (N.L.); 2 National Nanotechnology Center (NANOTEC), National Science and Technology Development Agency (NSTDA), Pathum Thani 12120, Thailand; jeeranan.non@nanotec.or.th; 3 Center of Excellence on Petrochemical and Materials Technology, Department of Chemical Engineering, Kasetsart University, Bangkok 10900, Thailand; fengttwi@ku.ac.th; 4 Research Network of NANOTEC-KU on NanoCatalysts and NanoMaterials for Sustainable Energy and Environment, Kasetsart University, Bangkok 10900, Thailand

**Keywords:** deoxygenation, coconut oil, in-situ hydrogen, nickel–cobalt bimetallic nanoparticles, biorefinery

## Abstract

The catalytic deoxygenation of coconut oil was performed in a continuous-flow reactor over bimetallic NiCo/silicoaluminophosphate-11 (SAPO-11) nanocatalysts for hydrocarbon fuel production. The conversion and product distribution were investigated over NiCo/SAPO-11 with different applied co-reactants, i.e., water (H_2_O) or glycerol solution, performed under nitrogen (N_2_) atmosphere. The hydrogen-containing co-reactants were proposed here as in-situ hydrogen sources for the deoxygenation, while the reaction tests under hydrogen (H_2_) atmosphere were also applied as a reference set of experiments. The results showed that applying co-reactants to the reaction enhanced the oil conversion as the following order: N_2_ (no co-reactant) < N_2_ (H_2_O) < N_2_ (aqueous glycerol) < H_2_ (reference). The main products formed under the existence of H_2_O or glycerol solution were free fatty acids (FFAs) and their corresponding C_n−1_ alkanes. The addition of H_2_O aids the triglyceride breakdown into FFAs, whereas the glycerol acts as hydrogen donor which is favourable to initiate hydrogenolysis of triglycerides, causing higher amount of FFAs than the former case. Consequently, those FFAs can be deoxygenated via decarbonylation/decarboxylation to their corresponding C_n−1_ alkanes, showing the promising capability of the NiCo/SAPO-11 to produce hydrocarbon fuels even in the absence of external H_2_ source.

## 1. Introduction

Hydrotreating is a typical process in the production of petroleum fuels from fossil crude oils using active sulfided catalysts, e.g., supported NiMo, CoMo, and NiW, operated under hydrogen atmosphere [1]. However, in order to secure economic feasibility as well as the environmental sustainability, many studies have been carried out to improve the process by finding new kinds of alternative resources, applying an inert ambient to the process, and/or using non-sulfided catalysts [2,3,4]. Nowadays, renewable oils, animal fats, and biodiesel from low-grade oils/fats are considered as alternative sources for hydrocarbon fuel production [5,6,7]. Lately, an upgrading of hydrocarbon fuels, which mostly consists of linear alkanes, to higher fraction of branched alkanes for the use as jet fuel-like hydrocarbons has received considerable attention because the demand of fuel for the aviation sectors has been increasing.

Principally, jet fuel-like hydrocarbon is produced from renewable oils through well-known processes where the oxygen in the oil’s molecules is removed by hydrodeoxygenation followed by hydro-isomerization/cracking, and distillation as the last step. To this procedure, a huge amount of hydrogen is required. For example, one molecule of triglyceride (TG) in vegetable oil reacts with twelve molecules of hydrogen gas (H_2_) for hydrodeoxygenation (HDO), six molecules of H_2_ for decarbonylation (DCO), or three molecules of H_2_ for decarboxylation (DCO_2_) route to form three molecules of alkanes [8,9]. The H_2_ consumption for the catalytic deoxygenation via those three major pathways is in the order of HDO > DCO > DCO_2_ [10]. Therefore, the DCO/DCO_2_ pathway has been more widely studied due mainly to its less hydrogen consumption. Specifically, three molecules of H_2_ are initially used for breaking a TG molecule into propane and free fatty acids (FFAs). Then, FFAs will further convert to hydrocarbons via DCO_2_ reaction without the need of hydrogen source. To promote the deoxygenation rate, well-designed non-sulfided catalysts are essential.

Hollak et al. [11] studied the catalytic activity of Pd/C catalyst for HDO of triglycerides (TGs) and FFAs. The results showed that even though the hydrogen produced in-situ from glycerol reforming can help for hydrocarbon product formation, the main products were still long-chain unsaturated hydrocarbons. This could be because the hydrogen production from glycerol using Pd/C catalyst was rather low. Similarly, Chiapper et al. [12] found that unsaturated hydrocarbon products were also the most selective in the deoxygenation of coconut oil and palm kernel oil over PtSnK/SiO_2_, even though mild hydrogenation treatment was performed to saturate mono- and polyunsaturated bonds in TGs prior to deoxygenation. A comparison of three catalysts, i.e., 1 wt% Pt/C, 5 wt% Pd/C, and 20 wt% Ni/C, was studied by Morgan and co-workers [13] for the deoxygenation of TGs (tristearin, triolein, or soybean oil) under N_2_ atmosphere. The supported non-noble Ni metal was found to provide significantly higher TGs conversion and fraction of C_8_–C_17_ compounds than the noble Pd and Pt metals. Miao et al. [14] also attempted to convert palmitic acid through non-noble Ni metal supported on ZrO_2_ with low-pressure or without external supply of H_2_ in the presence of water. The conversion of palmitic acid was significantly improved, and the results showed that hydrogenolysis promoted palmitic conversion and DCO occurred as the major reaction. Although non-noble Ni metal can be efficient to drive the reaction under H_2_ atmosphere, about 20 wt% of Ni loading was needed in their research. Apart from the widely used Ni-based catalysts, Co-based catalysts also have been reported providing high yield of hydrocarbons from deoxygenation of TGs (triolein, ceiba oil, and sterculia oil) under inert N_2_ flow condition via DCO_2_ pathway [15]. Moreover, it is well-documented that lower acidity of Co than Ni may assist lowering of catalyst coking and subsequent deactivation. Thus, Co shows potential to be addressed as an alternative or a component in Ni-based bimetallic catalysts.

The H_2_ produced from glycerol aqueous-phase reforming (APR) is of interesting to utilize for the in-situ deoxygenation of TG molecules. In 2002, the pioneer work [16] applied glycerol into the system to generate in-situ H_2_ for the reaction. However, the hydrogen production is always accompanied by several side reactions, such as methanol decomposition, water gas shift reaction, and methanation [17,18,19,20]. Therefore, the hydrogen yield is highly related to the performance of the catalysts which actively involves for the C–C, C–H, and C–O bonds scission, especially the cleavage of the C–H bond. The development of bimetallic catalysts such as Ni-Cu [17,18] or Ni-Co [19] is an efficient way to control the selectivity as the second metal can affect metal particle sizes or strengthen metal-support interaction to improve water gas shift reaction or inhibit methanation reaction.

Previously, Zhong and co-worker [21] studied the in-situ hydrogen production from high temperature water with Ni catalysts. In the system, high temperature water, with weak hydrogen bonds, was oxidized by active reductant metals as following; M + H_2_O → H_2_ + M_x_O_y_. Those results suggested that co-feeding with glycerol and water is clearly proved to be a facile hydrogen source for the deoxygenation of TGs to hydrocarbon fuels [21,22].

In this work, we studied the deoxygenation of TG to hydrocarbon alkanes via catalytic deoxygenation (under N_2_ ambient) and in-situ HDO (by H_2_O or glycerol addition) in comparison with conventional HDO (under H_2_ ambient) to yield oxygen-free hydrocarbons. The NiCo (about 10 wt% metal loading) supported on commercially available silicoaluminophosphate-11 (SAPO-11) was used as the deoxygenation catalyst. SAPO-11 is a molecular sieve material with 10-ring porous structure that has been extensively utilized due to its stability and potential activity in many catalytic processes [23,24]. The catalytic hydro/deoxygenation reactions of medium-chain (mostly C_12_) TGs in coconut oil, without hydrogenation treatment prior to deoxygenation, were examined to describe the reaction pathway via the degree of oxygen removal and product distribution, depending on the variable feed molecules. To the best of our knowledge, it is reported for the first time that SAPO-11-supported NiCo nanoparticles could directly convert coconut oil to jet fuel-like hydrocarbons with the in-situ hydrogen donors.

## 2. Materials and Methods

### 2.1. Materials

Commercial coconut oils were obtained from local companies in Thailand. Analytical grade glycerol (C_3_H_8_O_3_, 99% purity) was obtained from Fisher Scientific, Fair Lawn, NJ, USA. Hydrogen gas (H_2_, 99.99% purity) and nitrogen gas (N_2_, 99.5% purity) were obtained from S.I. Technology Co., Ltd., Bangkok, Thailand. SAPO-11 powder, used as the catalyst support, was purchased from ACS Material, Pasadena, CA, USA. Deionized (D.I.) water (18.2 MΩ·cm, at 25 °C) was produced by water purification machine in laboratory. Hydrocarbon standards (C_8_–C_18_) for calibration were purchased from Sigma-Aldrich, Buchs, Switzerland. Analytical reagent grade of nitrate salts of nickel (Ni(NO_3_)_2_·6H_2_O) and cobalt (Co(NO_3_)_2_∙6H_2_O) were purchased from Ajax FineChem, Taren Point, NSW, Australia.

### 2.2. Catalyst Preparation

The catalyst was prepared by co-impregnation technique by dissolving nitrate salts of Ni and Co in D.I. water. Then, SAPO-11 powder was further added to obtain 5 wt% loading of each metal. Mixing as well as drying were employed using a magnetic stirrer hot plate at 85 °C for 3 h and transferred to vacuum oven at the same temperature for overnight. The resultant powder was calcined in static air at 500 °C for 5 h. The final sample was denoted as NiCo/SAPO-11. Before catalytic testing, the catalyst powder was pelleted, sieved into 0.5–1.0 mm. in size.

### 2.3. Catalyst Characterization

Crystalline phase of the prepared catalyst was analysed by X-ray diffraction (XRD) measurement using an X-ray diffractometer (D8 ADVANCE, Bruker, Ltd., Karlsruhe, Germany) with Cu Kα radiation (wavelength of 1.5406 Å). The diffraction angle was scanned from 2θ of 5° to 80°, with steps of 0.5°/min and counting at 0.02 s/step. The specific surface area and pore structures of the samples were calculated by the Brunauer–Emmett–Teller (BET) and the density functional theory (DFT)/Barrett–Joyner–Halenda (BJH) methods, respectively, via nitrogen adsorption–desorption technique (Quantachrome Autosorb-iQ3, Boynton Beach, FL, USA). The total pore volume was measured at the relative pressure (P/P0) of 0.98. Before measurement, the sample of approximately 0.2 g was outgassed at 120 °C for 3 h under helium flow. Total acidity and strength of acid sites of catalyst were evaluated by ammonia (NH_3_)-temperature programmed desorption (NH_3_-TPD) carried out using Quantachrome Chemisorption Analyzer ChemStar TPX Series (Boynton Beach, FL, USA). Scanning electron microscopy (SEM) analysis was performed with a S-3400N (Hitachi-Science & Technology, Berkshire, UK), operated at 5 kV. Transmission electron microscopy (TEM) images were acquired by JEOL-JEM-2100Plus instrument (Akishima, Tokyo, Japan) equipped with an energy dispersive X-ray spectrometer (EDS). The TEM machine was operated at an accelerating voltage of 200 kV. Before measurement, the sample was reduced at 700 °C in a flow of H_2_ for 2 h. The obtained powder was grounded then dispersed in ethanol, followed by sonication for 30 min to make the particle well-dispersed, and dropped onto a gold grid.

### 2.4. Catalytic Deoxygenation Testing in Continuous-Flow Fixed-Bed Reactor

**Figure 1:**
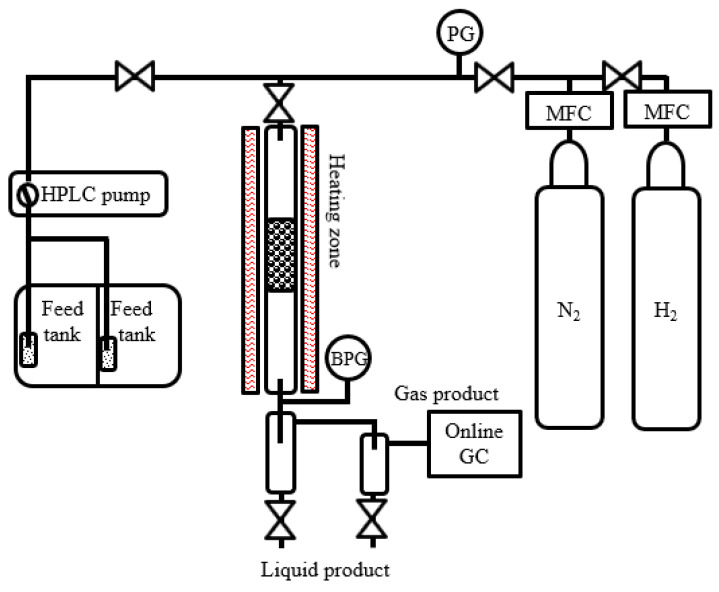
Schematic diagram of continuous-flow reactor system for catalytic testing.

The deoxygenation reaction was operated in a custom-made continuous-flow trickle-bed reactor made of the stainless steel 316, with an external diameter of 1.28 cm, internal diameter of 1 cm, and length of 70 cm. The catalyst bed was located at the middle of the fixed-bed reactor and held in position with quartz wool plugs at uniform temperature position (confirmed by inside thermocouples inserted in the catalyst bed). The reaction in the continuous system consists of a feed tank, a reactor unit, and a gas–liquid product separation unit, as shown in Figure 1. The temperature was controlled by using electrical furnace with two positions of temperature controller. A K-type thermocouple was inserted to contact the catalyst bed for temperature monitoring. The pressure of the reactor was manually controlled by using back pressure regulator (BPG) for two-phase flow. High-performance liquid chromatography (HPLC) pump and mass flow controller (MFC) were used to control the flow rate of liquid and gas feed, respectively. 

In a typical run, 4 mL of catalyst was loaded into a reactor and reduced with 50 cm^3^/min of pure H_2_ at 650 °C for 2 h. After the catalyst reduction step, the continuous reaction system was immediately flushed with reaction gas (N_2_ or H_2_) and set up to the desired temperature under pressurized condition. Liquid feeds—coconut oil, water, and glycerol solution—were separately introduced by two-suction line in the HPLC pump. 

Four different experimental practices were performed as follows:

Practice #1 was designed as a reference experiment for the purpose of investigating the catalytic performance on HDO of feed oil over NiCo/SAPO-11 catalyst. The reaction was performed at 330 °C under 50 bar of H_2_ atmosphere, and liquid feed (i.e., coconut oil) was introduced into the reactor with liquid hourly space velocity (LHSV) of 1 h^−1^. 

Practice #2 aimed to investigate the catalytic performance on the deoxygenation with absent H_2_. The procedure was similar to that described in practice #1, except the operating gas which was N_2_.

Practice #3 aimed to investigate the catalytic performance on the coupling deoxygenation of feed oil with in-situ H_2_ production by glycerol-APR. In short, the feed was introduced into the reactor by two-suction lines in HPLC pump, one for oil feed (80% by vol.) and one for glycerol solution feed (20% by vol.). The glycerol solution of 36 wt% glycerol in water (140 g of glycerol in 250 g of water) which was 0.1 mole ratio of glycerol: water) was used at a total LHSV of 1 h^−1^.

Practice #4 aimed to investigate the catalytic performance on the solvent-free HDO by adding 5% by volume of water into the reaction.

### 2.5. Liquid and Gaseous Product Analysis

Two-phase back pressure regulator was used to reduce a mixed-phase flow streams from high pressure into ambient before they went to a liquid–gas phase separator placed downstream. The liquid products were analysed by gas chromatography–mass spectrometry (GC–MS, Shimadzu GCMS-QP2020, Kyoto, Japan) equipped with a capillary column (DB-1HT, 30 m × 0.32 mm × 0.1 µm) to inform the quantitative/qualitative data of FFAs, hydrocarbons, and other intermediate products. Detailed information of this method is applied elsewhere [25,26]. Briefly, 10 µL of sample was injected into the injection port in a split mode with the inlet temperature of 340 °C. In a GC oven, the oven temperature was kept at 40 °C for 5 min then increased to 240 °C with a rate of 15 °C/min, then ramped by 8 °C/min to 370 °C, which was maintained for another 15 min. The total analysis run time was about 50 min. In the MS zone, the temperatures of ion source and interface were held at 250 °C during the analysis. The m/z was scanned from 2 to 500. The calibration curves of alkane standards (n-C_8_ to n-C_18_) were used to quantify their weight in the liquid products, and the response factor for isomer products was assumed to be equal to its corresponding n-alkanes. For other oxygenated intermediates, the calculation was referenced by the same type of known standards. 

Gas phase was analysed using an online GC equipped with two columns (molecular sieve 5A and Porapak Q), thermal conductivity detector (TCD) and flame ionization detector (FID). The GC was calibrated for all of the gaseous products obtained, including CO, CO_2_, CH_4_, C_2_H_6_, and C_3_H_8_.

Feed conversion and product selectivity were calculated based on mass balance. The catalytic performance was evaluated according to oil conversion (Equation (1)), liquid hydrocarbon yield (Equation (2)), selectivity to liquid hydrocarbon product (Equation (3)), and selectivity to gas species (Equation (4)):

Oil conversion (%) = ((mass of oil fed − mass of oil remaining)/(mass of oil fed)) × 100,(1)

Liquid hydrocarbon yield (%) = (mass of liquid hydrocarbon produced/mass of oil fed) × 100,(2)

Selectivity to liquid hydrocarbon product (%) = (mass of each liquid hydrocarbon product/mass of liquid hydrocarbon product in total) × 100,(3)

Selectivity to gas species (%) = (mass of each gas species/mass of gas in total) × 100,(4)

Contribution percentages of the HDO (Equation (5)) and DCO/DCO_2_ (Equation (6)) pathways were calculated based on the total moles of n-alkanes with even numbers or odd numbers of carbon atoms in the liquid product as follows:

HDO (%) = (mass of C_8_, C_10_, C_12_, C_14_, C_16_, and C_18_ in product/mass of feed) × 100, (5)

DCO+DCO_2_ (%) = (mass of C_9_, C_11_, C_13_, C_15_, and C_17_ in product/mass of feed) × 100, (6)

## 3. Results and Discussion

### 3.1. Catalyst Characterization

**Figure 2:**
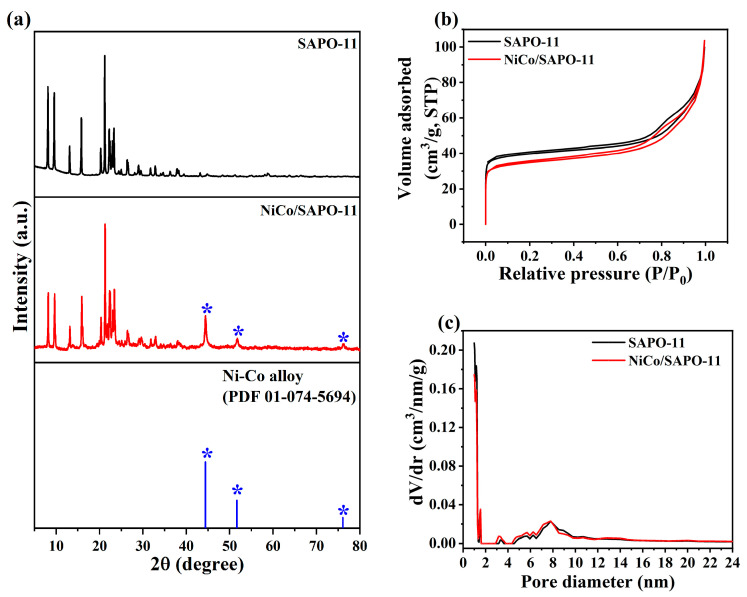
(**a**) XRD patterns, * 2θ = 44.41°, 51.78°, and 76.09° (powder diffraction file (PDF) for Ni-Co alloy (01-074-5694)); (**b**) N_2_ adsorption-desorption isotherms; and (**c**) pore-size distributions of SAPO-11 and the reduced NiCo/SAPO-11 catalyst.

The XRD pattern of NiCo/SAPO-11 (Figure 2a) suggested an alloy formation between Ni and Co, matched with PDF 01-074-5694, in accordance with Huynh and co-workers [27] who prepared bimetallic Ni-Co supported on different acidic materials used in Phenol HDO. Figure 2b,c show porous characteristics of the NiCo/SAPO-11 and SAPO-11 support. The obtained isotherms for all samples could be classified as type IV with hysteresis loops at 0.4 < P/P_0_ < 0.8 of H4 type [28]. These results showed that samples possess mesopore structures [29]. High adsorption of N_2_ appeared in the low relative pressure range, and the hysteresis loops were detected, suggesting the existence of both micropores and mesopores. The pore size distribution plots calculated by DFT method revealed two types of peaks with narrow micropores (high intensity at pore diameter < 2 nm) and mesopores (broad peak at pore diameter ≥ 2 nm) at approximately 0.98 and 7.78 nm, respectively. Upon NiCo loading, the BET surface area and pore volume of NiCo/SAPO-11 were ~134.4 m^2^/g and ~0.140 cm^3^/g, respectively, which were slightly lower than those of pristine SAPO-11 (~154.7 m^2^/g and ~0.139 cm^3^/g, respectively).

**Figure 3:**
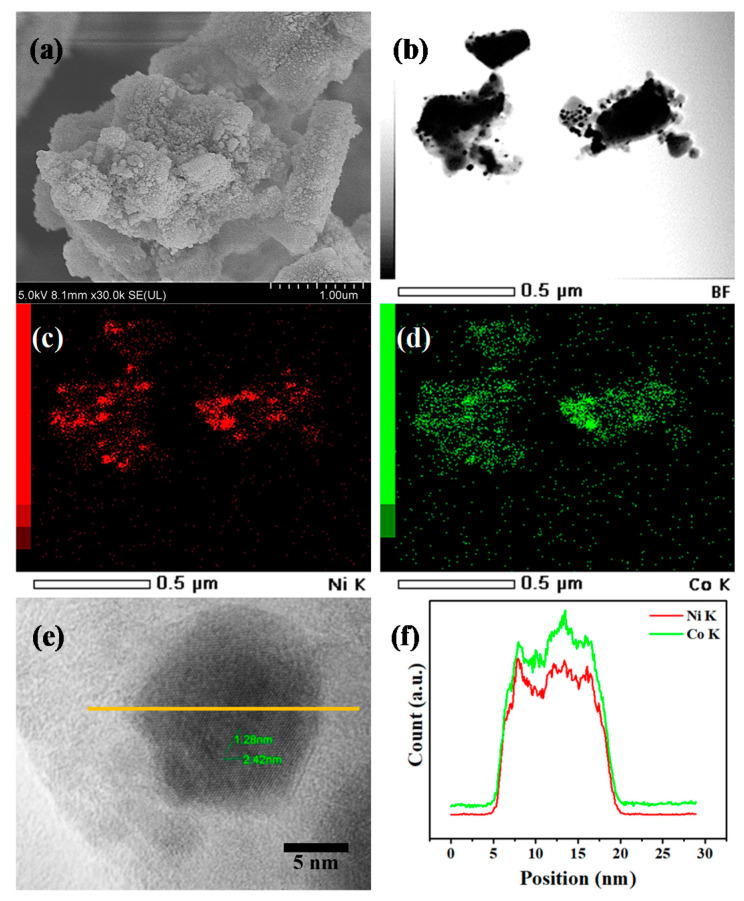
(**a**) Scanning electron microscopy (SEM) image; (**b**) transmission electron microscopy (TEM) image; (**c**,**d**) corresponding energy dispersive X-ray spectrometer (EDS) elemental mappings of Ni and Co; (**e**,**f**) EDS line profile of the NiCo/SAPO-11 catalyst.

The SEM image (Figure 3a) indicated that 10 wt% metal loading does not change the microstructure of SAPO-11 support, which is normally rod-like and has non-smooth surface. The bright field TEM image (Figure 3b) of the catalyst clearly shows Ni-Co nanoparticles, presented in black spots, in a spherical shape dispersed on the support, presented in the dark grey area. The metal particle size, estimated by measuring at least 100 particles, was about 9–18 nm. This range is consistent with San-Jose-Alonse and co-workers [30], who varied the proportion of Ni:Co. They showed that the particle size of samples prepared by impregnation was ~14.1 nm. (for Co), ~8.6 nm. (for NiCo), and ~7.0 nm. (for Ni). It is evidenced by EDS elemental mappings (Figure 3c,d) of the corresponding TEM image (Figure 3b) that Ni and Co species appeared at identical areas of the bimetallic particles, possibly due to the alloy structure formation, in good agreement with previous XRD results (Figure 2). Figure 3e,f display the high-resolution TEM (HRTEM) image of a single NiCo nanoparticle and its corresponding EDS line profile, respectively. These results confirm the co-existence of Ni and Co with comparable content of each metal, which was consistent with the analysis result from an inductively coupled plasma–mass spectrometry (ICP-MS) (results not shown). Additionally, lattice spacings of around 1.28 and 2.42 nm which were rotated by 53° were evidenced [31]. Evaluated by the NH_3_-TPD technique, total acidity of the SAPO-11 was 0.456 mmol/g, whereas NiCo/SAPO-11 catalyst showed lower values at 0.338 mmol/g (results not shown), coincided with the lower surface area of the supported catalyst.

### 3.2. Feed Compositions

Table 1 shows fatty acid compositions directly evaluated by GC–MS. It is seen that C_12_, C_16_, and C_18_ fatty acids are major components in coconut oil, with oxygen content ~13 wt%, the ratio of saturated/unsaturated FFAs ~3 times, which might induce different product distributions. The molecular weights for further calculation were 779.7 g/mol for coconut oil. 

### 3.3. Hydrogen Production from Glycerol Aqueous-Phase Reforming (Gly-APR) over NiCo/SAPO-11 

As of the complexity during the glycerol-APR process, the reactions of dehydration, dehydrogenation and hydrogenolysis could possibly take place on NiCo/SAPO-11 since the fact that this bifunctional catalyst comprises two main catalytic sites; (1) metal sites prefer to dehydrogenation and (2) acid sites prefer dehydration. Table 2 summarizes the main products such as propylene glycol (C_3_H_8_O_2_), ethylene glycol (C_2_H_6_O_2_), acetaldehyde (C_2_H_4_O), 1-propanol, 2-methyl- (C_4_H_10_O), 2-propanone, 1-hydroxy- (C_3_H_6_O_2_), and ethanol (C_2_H_5_OH) obtained in the condensable phase, which can be confirmed by the fact that side reactions occurred. The most abundant liquid product was ethylene glycol (~17.8%), followed by propylene glycol (~11.0%), and 2-propanone, 1-hydroxy- (~10.5%). In addition, trace amounts of 1-propanol, 2-methyl-, acetaldehyde, and ethanol could be detected (~7.1%). These results show a good agreement with the literature in that the same intermediates were reported [32,33]. The comparatively higher selectivity to ethylene glycol indicated that the primary hydroxyl group of glycerol is eliminated by the dehydrogenation pathway. However, the formation of propylene glycol and 2-propanone, 1-hydroxy- indicated that both pathways of the dehydration/hydrogenolysis were presented on Lewis sites of the catalyst. It also points out the high C–C, C–H cleavage activity of catalyst, accompanied by an increased production of hydrogen. As NiCo/SAPO-11 catalyst was identified to be of weak and medium strength acid sites (i.e., Lewis sites), this would lead to the formation of oxygenated hydrocarbons in the liquid phase (i.e., acetaldehyde, 1-propanol, 2-methyl-, 2-propanone, 1-hydroxy-, and ethanol), suggesting that Lewis sites are involved in C–O cleavage. The result also signifies that dehydrogenation, which competes with dehydration/hydrogenolysis, was significantly favoured over bifunctional NiCo/SAPO-11 catalyst.

Overall, the reaction pathways could be proposed that the reaction involves the C–O bond scission, followed by hydrogenation which consumes H_2_. Therefore, a part of H_2_ could be consumed and resulted in limited hydrogen production. The formation of ethylene glycol generated from dehydrogenation would support this assumption. 

Table 3 shows the main components of the gaseous products: CH_4_ > CO > CO_2_ > H_2_, respectively. As mentioned above, H_2_ yield was hardly maintained because H_2_ was consumed by side reaction (in liquid phase). Notably, the formation of CH_4_ also confirmed that H_2_ was used in CO/CO_2_ methanation (CO + 3H_2_ ↔ CH_4_ + H_2_O, ∆H_298_ = −206 kJ/mol and CO_2_ + 4H_2_ ↔ CH_4_ + 2H_2_O, ∆H_298_ = −164.94 kJ/mol) [33]. Generally, Ni species prefer to generate more CO. However, the amount of CO formed was higher than that of CO_2_, suggesting that bimetallic NiCo/SAPO-11 catalyst was favourable to the decomposition of methanol.

Co-based catalysts have been reported as less effective materials for the reforming of methanol [34,35]. However, Xue and co-worker [36] promoted Co into NiCu/Al catalyst for methanol reforming. The increased H_2_ amount showed that Co could promote the reaction by enhancement of water gas shift reaction. Papadopoulou and co-worker [37] investigated the adsorption of methanol on Co-based catalysts. TPD results indicated that the number of active sites provided the catalyst to be able to adsorb or decompose methanol molecules. In Table 3, methanol was not detected among the condensable phase products suggesting the possible reactions of methanol reforming (CH_3_OH + H_2_O ↔ CO_2_ + 3H_2_) or methanol decomposition (CH_3_OH ↔ CO + 2H_2_) over NiCo/SAPO-11 catalyst. This behaviour coincided with the synergistic effects of Ni-CO alloy in a NiCo/SAPO-11 catalyst in a positive way for H_2_ production.

### 3.4. Catalytic Performance of NiCo/SAPO-11 over In-Situ HDO of Coconut Oil

**Figure 4:**
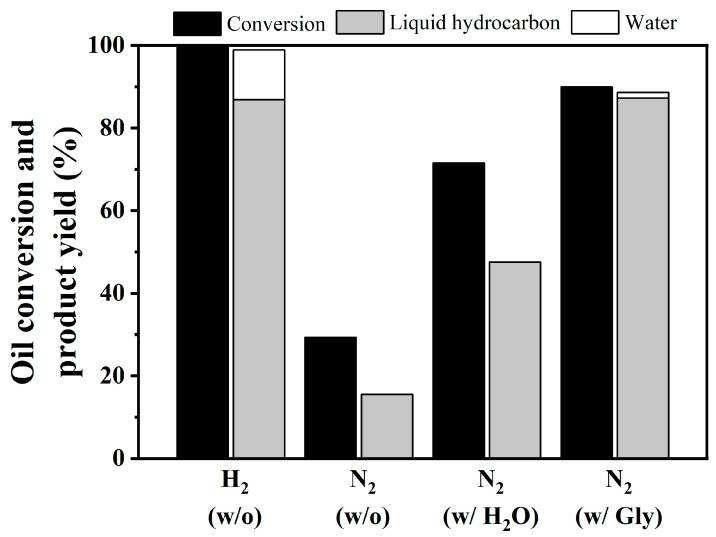
Oil conversion and product yield of coconut oil deoxygenation under different atmospheres with (w/) and without (w/o) co-reactant feeds. (Condition: Coconut oil, LHSV of 1 h^−1^, 330 °C, and 50 bar of reaction gas, Gly = glycerol solution).

Figure 4 displays the results of coconut oil HDO according to the change of co-reactant feed at 330 °C, LHSV 1 h^−1^, and 50 bar of reaction gas over 4 mL of NiCo/SAPO-11 catalyst. Under H_2_ ambient, the catalyst exhibited high activity with 100% conversion, while more than 85% of coconut oil conversion could yield the liquid product. Under an inert N_2_ atmosphere, the conversion was 30% and liquid product accounted for 15%. With the help of co-reactants, H_2_O and aqueous glycerol, the conversion increased to 70% and 90%, whereas liquid product yield also increased to 47% and 87%, respectively. Compared to the conventional H_2_ ambient, the results verified that the presence of H_2_O and glycerol helped to promote both coconut oil conversion and product yield. In addition, the experimental practices applying H_2_ below 50 bar (i.e., 30 bar) with and without glycerol solution were also performed. Even though 100% conversion was also achieved, hydrocarbon liquid yields were dropped to 65–67%, compared with using either 50 bar H_2_ without glycerol (Practice #1) or 50 bar N_2_ with glycerol solution (Practice #3). This could be because the existence of H_2_O under H_2_ ambient caused side reaction. Thus, the glycerol did not clearly selective to glycerol reforming. 

**Figure 5:**
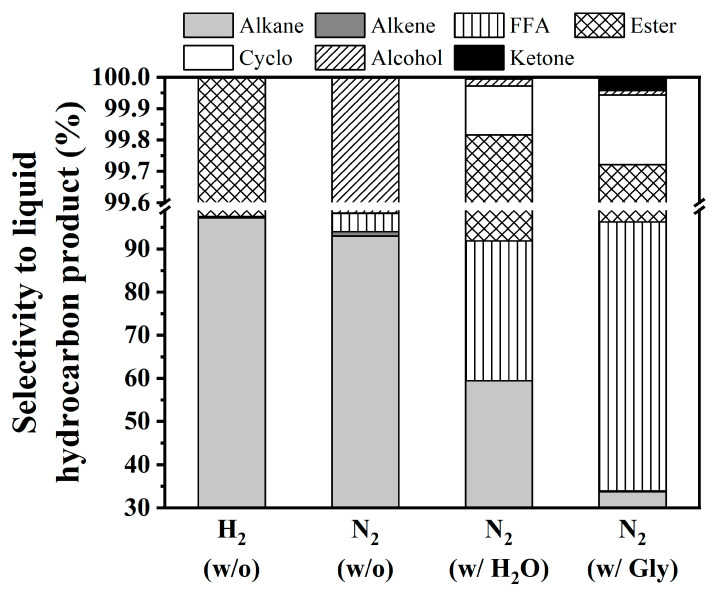
Distribution of liquid hydrocarbon products of coconut oil deoxygenation under different atmospheres with (w/) and without (w/o) co-reactant feeds. (Condition: Coconut oil, LHSV of 1 h^−1^, 330 °C, and 50 bar of reaction gas, Gly = glycerol solution).

**Figure 6:**
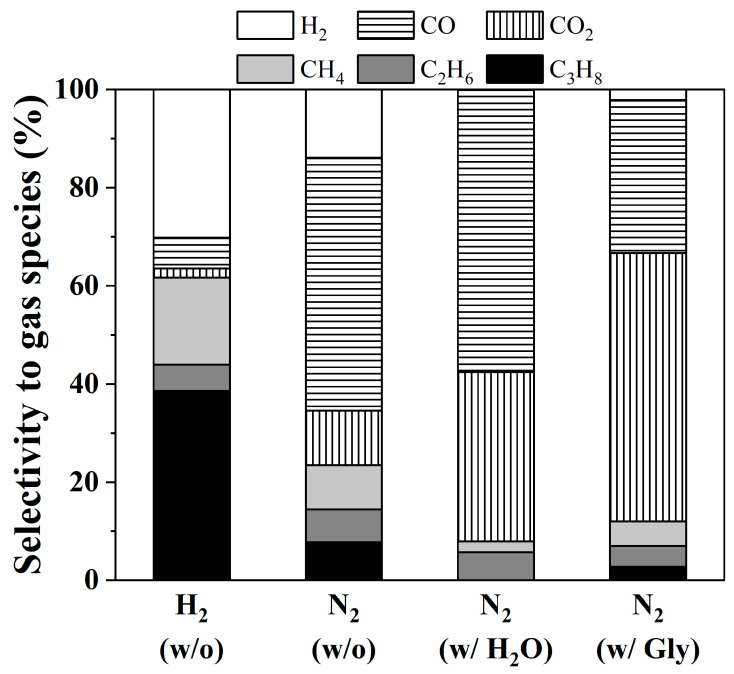
Distribution of gas species of coconut oil deoxygenation under different atmospheres with (w/) and without (w/o) co-reactant feeds. (Condition: Coconut oil, LHSV of 1 h^−1^, 330 °C, and 50 bar of reaction gas, Gly = glycerol solution).

Figure 5 shows the liquid product distribution over NiCo/SAPO-11 catalyst. With the help of H_2_, the catalyst could provide excellent selectivity to alkanes at 97%. Several intermediates (i.e., ketones, aldehydes, alcohols, cycloalkanes esters, FFAs, and alkenes) were detected in the liquid phase, while the C_3_H_8_, H_2_, CO, and CO_2_ were mainly formed in the gas phase. However, the product distribution was altered (see Figure 6). Under N_2_ atmosphere, main products are alkanes (C_11_–C_18_) and a small amount of FFAs, alkenes, and some alcohols. This finding is consistent with Morgan and co-workers [13], who established the thermal deoxygenation of TGs without H_2_ over 1 wt% Pt/C, 5 wt% Pd/C, and 20 wt% Ni/C catalysts. However, the existence of fatty acid composition was an important intermediate for alkane formation. Long chains of alkenes and alcohols were produced via hydrocracking. It can be implied that H_2_ generated from cracking affected the hydrocarbon production [38]. In the presence of H_2_O, CO and CO_2_ were mainly formed, while propane was not found in the bulk of gas product (see Figure 6). It can be considered that FFAs are successfully produced by the TG hydrolysis reaction route rather than deoxygenation which requires H_2_ molecules. However, the produced FFAs will either undergo DCO_2_ or DCO without H_2_ gas.

As also shown in Figure 6, the existence of methane (CH_4_) showed the consumption of H_2_. As a result, in-situ H_2_ produced via the water gas shift reaction (CO + H_2_O ↔ CO_2_ + H_2_, $\Delta H_{298}^{0}$ = −41 kJ/mol) [37] circulated in the system. Interestingly, two major side products were detected: high molecular weight esters or fatty esters (such as dodecanoic acid, dodecyl ester) and acetate compounds (such as octacosyl acetate). The formation of fatty esters might be due to the dehydration reaction of FFAs and alkyl alcohols on acid sites. It is believed that large molecular weight hydrocarbons lead to the deactivation of the catalyst by blocking the catalyst pores and depositing as coke. In addition, with an acidic functionality, acetate can be obtained from dehydrogenation of alcohol couples with aldehyde under an inert atmosphere to yield an acetate [38].

In H_2_O, the amount of FFAs, originating from hydrolysis of TGs, was much higher. This is in accordance with the literature [26,39,40], in which the disappearing of C=O of esters (in the TGs molecules) and the obtaining of C=O of carboxylic acids (in the FFAs molecules) in the reaction by using H_2_O were reported. This result confirmed that hydrolysis was the dominant pathway. The presence of H_2_O accelerated and promoted the conversion of TG molecules to FFAs. There was no consumption of hydrogen for the hydrolysis of the TGs. However, the H_2_ could be further utilized to promote deoxygenation of FFAs (DCO or DCO_2_). Therefore, the formation of the desired hydrocarbons was very low. It is known that when there was a lack of H_2_, which inhibits the catalytic performance, the reaction underwent cracking, for example: C_10_H_22_ → C_3_H_6_ + C_7_H_16_. Initially, hydrogen is present in the system because of some hydrocarbon molecules rearranging itself into a cycloalkane or an aromatic hydrocarbon with the loss of H_2_—for example: C_6_H_14_ → C_6_H_12_ + H_2_—and thus after prolonged reaction times, when this reservoir was consumed, an enhanced formation of aromatic C_n-1_ hydrocarbons was observed. Those of FFAs will either undergo DCO_2_ or DCO. This interesting phenomenon showed that alkenes were formed using either N_2_ or H_2_O. In the latter case, there was enough hydrogen and the formation hydrocarbons was almost constant.

The initial in-situ H_2_ can be generated from glycerol reforming (C_3_H_8_O_3_ + 3H_2_O ↔ 3CO_2_ + 7H_2_, $\Delta H_{298}^{0}$ = +128 kJ/mol) [41] and glycerol decomposition (C_3_H_8_O_3_ ↔ 3CO + 4H_2_, $\Delta H_{298}^{0}$ = +250 kJ/mol) [42,43]. The formation of H_2_, CO, and CO_2_ (Figure 6) confirmed the utilization of glycerol co-feeding to generate in-situ H_2_. These H_2_ initially aid the triglyceride breakdown to form propane and FFAs. Unfortunately, the abundant FFAs still remained in the liquid product. It exhibited the insufficiency of in-situ H_2_ so that alkanes could not be considerably produced. However, this in-situ H_2_ helps to decrease the level of high molecular weight hydrocarbon compounds.

Table 4 shows the proportion of DCOx/HDO and CO_2_/CO. The ratio of DCOx/HDO declined when applying co-reactant as follows: N_2_ > aqueous glycerol > H_2_O. The results indicated that generated H_2_ had a significant impact on reaction pathways. At the initial of reaction with glycerol co-reactant, an abundance of generated H_2_ can break C–O bonds in TG molecules through the HDO reaction to produce propane and FFAs. However, considering the final gas product, the reaction prefers to promote via DCO_2_ which does not require H_2_. It can be said that the rate of breaking of the C–O bonds in FFA should be slower than the C–O bonds in TGs and C–C bonds in FFA itself. On the other hand, the reaction seems to contribute to DCO when using H_2_O co-reactant. These results indicated that the insufficient H_2_ at the beginning suppressed HDO. Once FFA was generated, H_2_ from water gas shift reaction promoted DCO pathway, consistent with other reports [10,14,42,43,44,45,46]. In all conditions, the selectivity to jet fuel range (C_8_–C_14_) was higher than that to diesel fuel range (C_15_–C_18_).

## 4. Conclusions

The catalytic deoxygenation of coconut oil was performed on NiCo/SAPO-11 nanocatalyst in a continuous-flow reactor for hydrocarbon production. Reaction condition in assistance with in-situ H_2_ produced from glycerol (50 wt% aqueous solution), without any external H_2_ source (under N_2_ ambient) and addition of H_2_O (5% by volume) was investigated on the deoxygenation reaction and product distribution. The results showed that under N_2_ atmosphere applying co-reactants H_2_O and glycerol significantly increased the oil conversion. The main products were FFAs and their corresponding C_n−1_ alkanes, whereas a small fraction of the intermediates was found. Considering FFA products, the addition of H_2_O aids the triglyceride breakdown into FFAs, and the addition of glycerol solution as the hydrogen donor is favourable for the initial hydrogenolysis into FFAs. Then, those FFAs can be either decarbonylated or decarboxylated to their corresponding C_n−1_ alkanes, in the fraction of jet and diesel fuels. The NiCo/SAPO-11 showed great potential in the production of advanced biofuels in the absence of an external H_2_ source.

## Figures and Tables

**Table 1:** Fatty acid composition of coconut oil.

Fatty Acid	Content (wt%)
Octanoic acid (C8:0)	0.9
Decanoic acid (C10:0)	3.3
Dodecanoic acid (C12:0)	30.1
Myristic acid (C14:0)	21.5
Palmitic acid (C16:0)	17.9
Stearic acid (C18:0)	5.2
Oleic acid (C18:1)	15.9
Linoleic acid (C18:2)	4.8
Eicosanoic acid (C20:0)	0.2
Eicosenoic acid (C20:1)	0.2
Saturated FFAs	79.1
Unsaturated FFAs	20.9
Oxygen	12.9

**Table 2:** The list of investigated intermediates and their selectivity in liquid product during the glycerol aqueous-phase reforming over NiCo/SAPO-11 catalyst according to the GC–MS analysis.

Liquid Product		Retention Time (min)	Selectivity (%)
Acetaldehyde	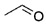	1.050	1.13
Ethanol	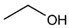	1.259	1.97
1-propanol, 2-methyl-	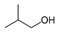	4.843	30.47
2-propanone, 1-hydroxy-	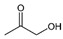	10.585	56.75
Propylene glycol	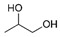	11.035	7.89
Ethylene glycol	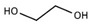	17.858	1.80

Reaction conditions: glycerol solution 36 wt%, and 4 mL catalyst, 230 °C, 30 bar N_2_.

**Table 3:** The yield of gas products during the glycerol aqueous-phase reforming over NiCo/SAPO-11 catalyst according to the GC–MS analysis.

Gas Product	Retention Time (min)	Yield (×10^−9^ mole)	Yield (×10^−5^ mol/mol Glycerol)
H_2_	1.78	0.0000084	0.00000024
CO	3.919	5.58528	1.5926
CH_4_	4.393	6.85314	1.95412
CO_2_	8.862	2.8679	0.81775

Reaction conditions: glycerol solution 36 wt%, and 4 mL catalyst, 230 °C, 30 bar N_2_.

**Table 4:** Summary reaction pathway and selectivity toward jet and diesel range fuels of the alkane products.

Feed	Co-Reactant	Selectivity (%)		DCO_x_/HDO ^3^	CO_2_/CO
		Jet Fuel ^1^	Diesel Fuel ^2^		
Coconut oil	H_2_	64.09	35.91	1.08	0.30
	N_2_	28.73	71.27	2.90	0.21
	H_2_O	62.58	37.42	1.05	0.59
	Glycerol	58.74	41.26	1.18	1.75

^1^ Jet fuel range = C_8_–C_14_ alkanes, ^2^ Diesel fuel range = C_15_–C_18_ alkanes, ^3^ DCO_x_ = DCO + DCO_2_.
